# Effect of long‐term maximum strength training on explosive strength, neural, and contractile properties

**DOI:** 10.1111/sms.14120

**Published:** 2022-01-15

**Authors:** Thomas G. Balshaw, Garry J. Massey, Thomas M. Maden‐Wilkinson, Marcel B. Lanza, Jonathan P. Folland

**Affiliations:** ^1^ Versus Arthritis Centre for Sport, Exercise and Osteoarthritis Loughborough University Leicestershire UK; ^2^ School of Sport, Exercise, and Health Sciences Loughborough University Leicestershire UK; ^3^ School of Sport and Health Sciences University of Exeter Exeter UK; ^4^ Academy of Sport and Physical Activity Faculty of Health and Wellbeing Collegiate Campus Sheffield Hallam University Sheffield UK; ^5^ Department of Physical Therapy and Rehabilitation University of Maryland Baltimore Baltimore US

**Keywords:** agonist muscle, antagonist muscle, rate of torque development, strength training, surface electromyography

## Abstract

The purpose of this cross‐sectional study was to compare explosive strength and underpinning contractile, hypertrophic, and neuromuscular activation characteristics of long‐term maximum strength‐trained (LT‐MST; ie, ≥3 years of consistent, regular knee extensor training) and untrained individuals. Sixty‐three healthy young men (untrained [UNT] *n* = 49, and LT‐MST *n* = 14) performed isometric maximum and explosive voluntary, as well as evoked octet knee extension contractions. Torque, quadriceps, and hamstring surface EMG were recorded during all tasks. Quadriceps anatomical cross‐sectional area (QACSA_MAX_; via MRI) was also assessed. Maximum voluntary torque (MVT; +66%) and QACSA_MAX_ (+54%) were greater for LT‐MST than UNT ([both] *p* < 0.001). Absolute explosive voluntary torque (25–150 ms after torque onset; +41 to +64%; [all] *p* < 0.001; 1.15≤ effect size [ES]≤2.36) and absolute evoked octet torque (50 ms after torque onset; +43, *p* < 0.001; ES = 3.07) were greater for LT‐MST than UNT. However, relative (to MVT) explosive voluntary torque was lower for LT‐MST than UNT from 100 to 150 ms after contraction onset (−11% to −16%; 0.001 ≤ *p* ≤ 0.002; 0.98 ≤ ES ≤ 1.11). Relative evoked octet torque 50 ms after onset was lower (−10%; *p* < 0.001; ES = 1.14) and octet time to peak torque longer (+8%; *p* = 0.001; ES = 1.18) for LT‐MST than UNT indicating slower contractile properties, independent from any differences in torque amplitude. The greater absolute explosive strength of the LT‐MST group was attributable to higher evoked explosive strength, that in turn appeared to be due to larger quadriceps muscle size, rather than any differences in neuromuscular activation. In contrast, the inferior relative explosive strength of LT‐MST appeared to be underpinned by slower intrinsic/evoked contractile properties.

**Abbreviations**
EMG: electromyography
ES: effect size
knee flexion EMG_MAX_
: EMG during knee flexion maximum voluntary torque
LT‐MST: long‐term maximum strength training
MVT: maximum voluntary torque
QACSA_MAX_
: maximum quadriceps femoris anatomical cross‐sectional area
QEMG/HEMG: quadriceps or hamstring EMG
QEMG_0‐50_/HEMG_0‐50_
: quadriceps/hamstring EMG during specific time period (ms) after EMG onset
RTD/RTD_0‐50_
: rate of torque development/RTD at specific time period (ms) after contraction onset
T/T_50_
: torque/torque at specific time (ms) after contraction onset
TPT: time to peak torque
Twitch/Octet T_50_
: torque at 50 ms after contraction onset during twitch/octet contractions
Twitch/Octet TPT: time to peak torque during twitch/octet contractions
UNT: untrained

## 1 INTRODUCTION

Explosive strength, the ability to increase force or torque rapidly from a low or resting level,^1^, ^2^, ^3^ is widely considered to be important for the performance of athletic tasks (ie, sprinting and jumping^4^) and daily living activities (standing from a seated position, brisk walking^5^). Furthermore, explosive strength is considered important in situations where the time to develop torque is limited, such as prevention of falls.^6^, ^7^ In contrast, maximum strength, the greatest force a muscle group can produce, is important for the performance of athletic tasks where force production is less constrained by time (ie, wrestling and rugby scrummaging^8^, ^9^) and activities of daily living (ie, ambulation) in older adult and patient populations.^10^, ^11^ The specificity or commonality of these two types of strength and their adaptations to resistance training remains to be fully understood.

We have previously found considerable specificity in the functional, and underpinning physiological adaptations, in response to training for explosive vs maximum strength during relatively short‐term training programs (4–12 weeks^12^, ^13^). Notably, explosive strength training with short, rapid contractions performed as explosively as possible was most effective at developing explosive strength (ie, rapid, explosive force production), and maximum strength training with sustained contractions at high loads, but no emphasis on generating force quickly, was superior for developing maximum strength.^12^, ^13^ Furthermore, the changes in neuromuscular activation also appeared to be specific to the type of training performed (ie, only explosive strength training increased explosive phase neuromuscular activation). In these isometric studies, perhaps surprisingly maximum strength training produced no benefits in early phase explosive force production (≤100 ms),^12^, ^13^ and this was also the case for short‐term isoinertial/dynamic maximum strength training.^14^, ^15^ Although isometric,^12^, ^13^ but not isoinertial,^14^, ^15^ MST increased late phase (>100 ms) explosive force production. Furthermore, due to increases in maximum strength in these studies, but no/minor increases in absolute explosive strength, relative explosive strength (ie, % of maximum voluntary torque [MVT]) decreased after MST.^12^, ^13^, ^14^, ^15^ This might be because the intrinsic contractile properties (ie, evoked force responses) showed a slowed timecourse independent of any changes in absolute force amplitude, principally longer evoked twitch and octet time to peak tension, and lower relative evoked explosive forces of the trained muscle after 12 weeks of maximum strength training, which was also the case following explosive strength training.^12^ Thus, slower intrinsic contractile properties and no improvement in explosive phase neuromuscular activation likely explain the minor (absolute) or negative (relative) changes in explosive strength after short‐term maximum strength training.^12^, ^13^ Therefore, the short‐term value of conventional maximum strength training for explosive strength development may appear limited.

On the other hand, maximum strength training progressively elicits a range of adaptations that are known determinants of explosive strength (eg, increased muscle size^14^, ^16^, ^17^ and maximum strength^3^, ^18^). Therefore, prolonged maximum strength training (ie, long term, >6 months) might be expected also to be beneficial for explosive strength, in contrast to the short‐term studies described above. While longitudinal intervention studies of several years’ duration may be impractical, cross‐sectional comparisons of chronically strength‐trained groups may be informative. Using this approach, we found that explosive power athletes that typically perform explosive strength training had substantially greater explosive strength (force after 50 ms of contraction: absolute +124%; relative +73% vs untrained controls)^19^ and these findings were recently corroborated.^20^ However, prior studies of individuals with extensive maximum strength training experience (ie, multiple years) have reported contradictory findings,^21^, ^22^ with maximum strength training individuals found to have both similar^21^ and higher^22^ explosive strength than control groups. Consequently, it remains unclear as to whether or not long‐term maximum strength training may be beneficial for explosive strength.

Therefore, the purpose of the current investigation was to compare long‐term maximum strength‐trained (LT‐MST, ie, multiple years of training to increase maximum strength) and untrained (UNT) individuals for: (i) knee extension voluntary explosive isometric strength throughout the rising torque‐time curve, in absolute and relative (to MVT) terms; and (ii) the primary physiological determinants of explosive strength, intrinsic contractile properties (ie, evoked octet and twitch torque) and neuromuscular activation assessed with surface EMG. It was hypothesized that LT‐MST individuals (vs UNT) would have higher absolute explosive strength but lower relative explosive strength (to MVT); slower evoked contractile properties; and equivalent neuromuscular activation during explosive contractions.

## 2 MATERIALS AND METHODS

### 2.1 Participants

A total of sixty‐three young, healthy males with no history of major, traumatic lower‐body injury provided written informed consent prior to their participation in this study that was approved by the Loughborough University Ethical Advisory Committee. Some data from these participants (specifically maximum strength and associated EMG, but not explosive strength or evoked contractile properties) were a component of a previous publication.^23^ The International Physical Activity Questionnaire [short format^24^] was used to assess the physical activity levels of all participants. The UNT group consisted of 49 participants (International Physical Activity Questionnaire: 2326 ± 1337 metabolic equivalent min/wk) who had not completed lower‐body resistance training for >18 months and were not undertaking any systematic physical training at the time of participation in this study. The resistance training history, participation in competitive sports, use of nutritional supplements, and androgenic‐anabolic steroids of potential LT‐MST participants were assessed by a detailed questionnaire that participants were asked to sign as an accurate record and where necessary a follow‐up verbal discussion. The LT‐MST group consisted of 14 participants (International Physical Activity Questionnaire: 5568 ± 1457 metabolic equivalent min/wk) who had been completing systematic heavy (ie, loads ≥70% of one repetition maximum) resistance training of the quadriceps for ≥3 years (mean ± SD, 4 ± 1 years, range 3–5 years) with the primary aim of developing maximum strength. Specifically, LT‐MST group participants had been completing several knee extensor exercises (typically consisting of: squat, lunge, step‐up, and leg press) within each individual lower‐body training session ~3 x/wk throughout their RT. Within the LT‐MST group, 7 participants’ sole systematic physical activity was RT, 5 participants were national level rugby union players, and 2 participants were competing in powerlifting/body building. This group had received variable coaching (technique and programming) support during their resistance training. Many individuals in the LT‐MST group reported regular use of nutritional supplements (eg, whey protein and creatine). Use of androgenic‐anabolic steroids was an exclusion criterion.

### 2.2 Overview

Participants completed a familiarization session involving unilateral isometric voluntary maximum and explosive contractions, as well as evoked contractions, while seated on a rigid custom‐built isometric knee extension/flexion dynamometer (as shown in Figure 6B of ^25^). Following familiarization duplicate, neuromuscular measurement sessions were conducted with the dominant leg at a consistent time of day for each individual (starting between 12:00 and 19:00, 7–10 days apart, and with the first session 3–7 days after familiarization). Neuromuscular measurement sessions involved recordings of isometric knee extension/flexion torque and surface EMG of the superficial quadriceps and hamstrings muscles during the same tasks and with the same dynamometer used for during familiarization. The primary outcome measures were explosive torque (absolute and relative to MVT), simultaneous quadriceps and hamstring EMG and evoked intrinsic contractile properties (assessed via twitch and octet contractions). Agonist, quadriceps, EMG was expressed in absolute terms (corrected for muscle‐electrode distance assessed with ultrasound, to reduce the confounding influence of adiposity) and also normalized to EMG at MVT. Muscle size was also assessed with a T1‐weighted 1.5T MRI scan of the thigh of each participant's dominant leg, within 7 days of the second neuromuscular measurement session, as an additional index of training status and morphological differences.

### 2.3 Torque and EMG recording

All contractions were completed with participants secured in a rigid custom‐made isometric dynamometer (knee and hip angles of 115**°** and 126°; 180° = full extension). Extraneous bodily movement was minimized by tightly fastening adjustable straps across the pelvis and shoulders. A reinforced canvas webbing strap (35 mm width) secured around the ankle was placed at ~15% of tibial length (distance from lateral malleolus to knee joint space), above the medial malleolus, positioned posterior and perpendicular to the tibia and in series with a calibrated S‐beam strain gauge (Force Logic, Swallowfield, UK). The analogue force signal from the strain gauge was amplified (x370) and sampled at 2,000 Hz using an external A/D converter (Micro 1401; CED Ltd., Cambridge, UK) and recorded with Spike 2 computer software (CED Ltd., Cambridge, UK). In offline analysis, force data were low‐pass filtered at 500 Hz using a fourth‐order zero‐lag Butterworth filter, gravity corrected by subtracting baseline force, and multiplied by lever length, the distance from the knee joint space to the center of the ankle strap, to calculate torque values.

Surface EMG was recorded from the superficial quadriceps (QEMG: rectus femoris; vastus lateralis; vastus medialis) and medial and lateral hamstring muscles (HEMG) using a wireless EMG system (Trigno; Delsys Inc., Boston, MA). The skin at specific locations was prepared (shaving, abrading, and cleansing with 70% ethanol) before single differential Trigno Standard EMG sensors (Delsys Inc., Boston, MA; bipolar fixed 1‐cm interelectrode distance) were positioned with adhesive interfaces. Individual sensors were attached over the superficial quadriceps femoris muscles at six separate sites (ie, two entirely independent sensors on each muscle to improve the reliability of EMG measurements^26^) at set percentages of thigh length (above the superior border of the patella) as follows: rectus femoris 65% and 55%; vastus lateralis 60% and 55%; and vastus medialis 35% and 30%. Individual sensors were also placed on the medial and lateral hamstring muscles at 45% of thigh length above the popliteal fossa. The location of hamstring EMG sensors was determined by palpating the borders of the biceps femoris long head and the medial hamstrings (semitendinosus and semimembranosus), respectively. Sensors were placed parallel to the presumed orientation of the underlying fibers. EMG signals were amplified at source (x300; 20‐ to 450‐Hz bandwidth) before further amplification (overall effective gain, x909) and sampled at 2,000 Hz via the same A/D converter and computer software as the force signal to enable data synchronization. In offline analysis, EMG signals were corrected for the 48‐ms delay inherent to the Trigno EMG system.

### 2.4 Neuromuscular Measurement session

To prepare for maximum, explosive and evoked contractions participants completed a warm‐up of their dominant leg (3 s knee extension contractions at 50% [x3], 75% [x3], and 90% [x1] of perceived maximum) with the same dynamometer used throughout the testing session. Measurements were then completed in the following order.

### 2.5 Knee extension isometric maximum voluntary contractions

Participants were instructed to “push as hard as possible” for 3–5 s during maximum voluntary contractions and rest for ≥30 s between a total 3–4 maximum voluntary contractions. Real‐time biofeedback was provided via a computer monitor in front of the participant displaying the torque‐time curve, and a horizontal cursor was used to indicate the greatest torque obtained within that session.^3^, ^27^ Intense verbal encouragement was provided during all maximum voluntary contractions. Knee extensor MVT was defined as the greatest instantaneous torque achieved during any maximum voluntary contraction during that measurement session. Root mean square EMG for a 500‐ms time window at MVT (250 ms either side) was calculated for each electrode site. Root mean square EMG from each quadriceps site was corrected for muscle‐electrode distance (see below), then averaged across the six sites to provide an overall absolute quadriceps EMG measurement during MVT production (QEMG_MVT_).

### 2.6 Knee extension explosive voluntary contractions

Ten explosive voluntary contractions were completed by each participant following the instruction to perform each contraction “as fast and hard as possible” for ~1 s, in order to exceed 80% MVT. Participants relaxed for ≥15 s between contractions. Contractions with pre‐tension or countermovement (ie, a change in baseline torque in either direction) of >0.34 Nm in the 300 ms prior to contraction onset were discarded. The three explosive efforts with the highest torque at 100 ms after contraction onset were analyzed in detail for torque and EMG. Voluntary explosive torque was measured at 25, 50, 75, 100, and 150 ms from contraction onset (T_25_, T_50_, T_75_, T_100_, and T_150_), before averaging across the three contractions. Explosive torque was also expressed relative to MVT to assess the ability of each participant to utilize their available torque production capacity. Consecutive rate of torque development (RTD) windows was calculated between 0–50 ms, 50–100 ms, and 100–150 ms both in absolute terms and expressed relative to MVT.

Root‐mean‐square EMG of each of the quadriceps and hamstring sensors during explosive contractions was measured over three consecutive time periods (ie, 0–50, 50–100, and 100–150 ms from EMG onset of the first quadriceps muscle to be activated [see below]). Measurements for each recording site were averaged across the three best explosive contractions. Quadriceps EMG measurements were then corrected for muscle‐electrode distance (see below). All the recording sites for each muscle group were averaged, to produce overall absolute quadriceps (QEMG_0–50_, QEMG_50–100_, QEMG_100–150_) and hamstring (HEMG_0–50_, HEMG_50–100_, HEMG_100–150_) values. Root‐mean‐square EMG values from each sensor during explosive knee extension contractions were also normalized to agonist EMG during maximum knee extension (quadriceps) or maximum knee flexion (hamstrings) before averaging across sensors to produce overall normalized quadriceps and hamstring EMG measurements. The ratio of torque produced at 50 ms after contraction onset between voluntary explosive contractions and evoked octet contractions (ie, Voluntary T_50_/ Octet T_50_ [see below]) was used as an additional measure of volitional neural efficacy.

During offline analysis, all torque and quadriceps EMG onsets were identified manually by visual identification using a systematic approach^19^, ^28^ considered to be more valid than automated methods.^28^ All manual visual onset identification was completed by one trained investigator. Torque and EMG signals were initially viewed on an x‐axis scale of 300 ms prior to the contraction and y‐axis scales of 0.68 Nm (torque) or 0.05 mV (EMG)^19^, ^28^ before viewing signals on a more sensitive scale to determine the instant of the last peak or trough before the signal deflected away from the envelope of the baseline noise. Torque and EMG variables measured from the three explosive contractions with the highest torque at 100 ms were averaged across these selected contractions.

### 2.7 Evoked twitch and octet contractions

The femoral nerve was electrically stimulated using a constant‐current variable‐voltage stimulator (DS7AH; Digitimer, Welwyn Garden City, UK), cathode probe (1‐cm diameter; Electro‐Medical Supplies, Wantage, UK), and anode electrode (7 x 10‐cm carbon rubber electrode; Electro‐Medical Supplies, Wantage, UK). The cathode and anode were both coated with electrode gel and secured to the skin using transpore tape while positioned over the femoral nerve in the femoral triangle and over the greater trochanter, respectively. The location of the cathode was first determined by delivering single electrical impulses (square wave pulses of 0.2‐ms duration, ≥12 s apart) to identify the position that produced the greatest twitch torque response to a constant sub‐maximum stimulus. The current intensity was increased in a stepwise manner until plateaus in peak twitch torque were reached. Finally, three supramaximal twitch responses were evoked (15 s apart) at a higher current (≥50%) to ensure supramaximal stimulation. The following variables were averaged across the three supramaximal twitch contractions: peak twitch torque (Twitch Peak T), twitch time to peak torque (Twitch TPT), absolute torque at 50 ms after contraction onset (Twitch T_50_), and torque at 50 ms expressed relative to Twitch Peak T (Relative Twitch T_50_).

After the twitch contractions during the second neuromuscular measurement session only, octet contractions (8 impulses at 300 Hz) were evoked at progressive currents (≥15 s apart) until plateaus in peak octet torque and peak octet RTD occurred. Three discrete pulse trains (≥15 s apart) were then delivered with a higher current (≥20% above that required to generate a plateau in octet peak torque and RTD, to ensure supramaximal stimulation) to evoke maximum octet contractions. Octet contractions of this type have been shown to evoke the maximum possible RTD from the muscle‐tendon unit^29^, ^30^ and thus provide an index of the maximum intrinsic contractile ability for explosive force production. The following variables were averaged across the three supramaximal octet contractions: peak torque (Octet Peak T), octet time to peak torque (Octet TPT), absolute torque at 50 ms after contraction onset (Octet T_50_), and torque at 50 ms expressed relative to Octet Peak T (Relative Octet T_50_). Because of the discomfort caused by the octet contractions, two participants from the LT‐MST group were unable to tolerate this measurement.

### 2.8 Knee flexion maximum voluntary contractions

Knee flexion maximum voluntary contractions were performed in the same manner as knee extension maximum voluntary contractions, except participants performed a series of warm‐up sub‐maximum knee flexion efforts and were instructed to “pull as hard as possible” for 3–5 s, rather than “push.” The greatest instantaneous torque achieved during any maximum voluntary contraction was identified and a root‐mean‐square EMG for a 500‐ms time window (ie, 250 ms either side of knee flexor MVT) was calculated for each hamstring electrode site. Root‐mean‐square EMG from each hamstring site was then averaged to provide an overall absolute hamstring EMG measurement during knee flexion MVT production (knee flexion EMG_MAX_).

### 2.9 Imaging session

#### 2.9.1 Muscle size

A 1.5 T MRI scan of the dominant leg was conducted with participants in the supine position at a knee joint angle of ~163° using a receiver 8‐channel whole body coil (Signa HDxt, GE). T1‐weighted axial slices (5 mm thick, 0 mm gap) were obtained between the anterior superior iliac spine and the knee joint space in two overlapping blocks. Oil‐filled capsules were secured with transpore tape to the lateral side of the participants’ thigh to allow alignment of the two blocks during manual segmentation analysis. MR images were analyzed by two trained investigators using Osirix software (version 6.0, Pixmeo, Geneva, Switzerland). The quadriceps muscles (rectus femoris, vastus lateralis, vastus medialis, and vastus intermedius) were manually outlined in every third image (ie, every 15 mm) starting from the most proximal image, in which the muscle first appeared. The image with the largest anatomical cross‐sectional area (ACSA) was defined as the maximum ACSA for each individual quadriceps muscle. The sum of the constituent quadriceps muscle maximum ACSAs was defined as maximum quadriceps ACSA (QACSA_MAX_).

### 2.10 Muscle‐electrode distance for correction of absolute agonist EMG amplitude

To correct for the confounding influence of the amount of tissue between the recording site and the muscle (ie, predominantly subcutaneous fat) on surface EMG amplitude, B‐mode ultrasonography images were recorded at each of the six sites where quadriceps EMG sensors were positioned. These images facilitated measurement of the distance between the skin surface and the peripheral surface of the muscle at each of the six EMG sites (ie, muscle‐electrode distance). Our previous work recommended muscle‐electrode distance correction in studies where systematic differences in muscle‐electrode distance (subcutaneous adiposity) might be expected between groups.^31^ Ultrasonography images were recorded (EUB‐8500 ultrasound machine; Hitachi Medical Systems UK Ltd, Northamptonshire, UK) using a B‐mode ultrasonography machine (EUB‐8500; Hitachi Medical Systems UK Ltd, Northamptonshire, UK) and a 9.2‐cm‐wide linear array transducer (EUP‐L53L) interfaced with a personal computer operating ezcap video capture software. The transducer was coated with water‐soluble transmission gel and placed perpendicular to the skin over the rectus femoris, vastus lateralis, and vastus medialis at the percentages of thigh length listed above for each quadriceps EMG sensor. Images were later imported into a public domain software (Tracker version 4.92; www.cabrillo.edu/~dbrown/tracker), and muscle‐electrode distance was measured by one trained investigator.

When agonist EMG measurements at MVT for all participants in both cohorts were pooled (ie, *n* = 63), there were inverse relationships between absolute EMG amplitude during MVT and muscle‐electrode distance for all quadriceps sensor locations (Pearson's product moment bivariate correlations, −0.628 ≤ r ≤ −0.477; [all] *p* < 0.001). Additionally, when comparing the two groups, quadriceps muscle‐electrode distance (ie, mean muscle‐electrode distance across all locations) was significantly less for LT‐MST (0.8 ± 0.2 cm) than UNT (1.0 ± 0.4 cm; independent samples t‐test *p* = 0.049; effect size = 0.61 “Moderate”). Consequently, all individual agonist EMG measurements during MVT production were corrected for muscle‐electrode distance by: (i) solving the polynomial relationship (second or third order) between agonist EMG amplitude and muscle‐electrode distance at that specific measurement site; (ii) calculating the difference between the actual (measured) and expected EMG (derived from polynomial) amplitude; and (iii) summating the individual's residual (actual EMG—expected EMG) absolute agonist EMG amplitude with the pooled group mean for actual absolute agonist EMG amplitude. EMG during the explosive contractions was then corrected/scaled in proportion to the correction applied to EMG at MVT (according to the ratio of actual EMG/expected EMG at MVT).

### 2.11 Data analysis and statistics

All torque and EMG measurements from the two neuromuscular measurement sessions were averaged to produce criterion values. All statistical analyses were performed using SPSS Version 26.0 (IBM Corp., Armonk, NY). Data are reported as means ± SD, and the significance level was set at *p* < 0.05. Tendencies (0.05 < *p* < 0.10) were noted only in instances where the effect size was greater than 0.50 and adjacent time points/periods also showed significant effects, increasing the likelihood of a genuine systematic effect. The Shapiro‐Wilk test was used to assess data normality across variables within each group. The majority of the variables were normally distributed within each group (UNT 58%; LT‐MST 85%). Consequently, parametric statistics were used for a consistent approach. Independent sample t‐tests were conducted to assess whether between group differences existed for: descriptive characteristics (ie, age, height, and body mass), maximum strength, muscle size, explosive strength (absolute and expressed relative to MVT), quadriceps and hamstring EMG during explosive contractions (absolute and normalized to agonist EMG during MVT production), and evoked twitch and octet torque variables. As previously detailed for between‐participant study designs^32^ effect size (ES) was calculated as: Difference in group means (largest minus smallest group mean) divided by the square root of the pooled SD. Pooled SD was calculated as: ([LT‐MST sample size ‐ 1] x [LT‐MST SD^2^] + [UNT sample size ‐ 1] x [UNT SD^2^]) ÷ ([LT‐MST sample size + UNT sample size] – 2). ES values were classified as follows: <0.20 “trivial,” 0.20 – 0.49 “small,” 0.50 – 0.79 “moderate,” or ≥0.80 “large.”

Between‐test session reliability of key measurements was assessed by pooling both groups of participants (ie, *n* = 63) using within‐participant coefficient of variation (CV_W_, [SD/mean] × 100) as a measure of absolute reliability.

## 3 RESULTS

### 3.1 Between‐test session reliability

Knee extension MVT displayed a mean CV_W_ values of 3.1%. Explosive knee extension torque at T_25_, T_50_, T_75_, T_100_, and T_150_ demonstrated mean CV_W_ values of 14.7%, 16.4%, 9.4%, 7.0%, and 5.1%, respectively. Corrected agonist EMG during explosive knee extension contractions had mean CV_W_ values of 19.1%, 13.6%, and 13.8% for QEMG_0‐50_, QEMG_50‐100_, and QEMG_100‐150_, respectively. Normalized hamstring EMG during explosive knee extension contractions demonstrated mean CV_W_ values of 40.8%, 30.2%, and 33.1% for HEMG_0‐50_, HEMG_50‐100_, and HEMG_100‐150_, respectively. Peak Twitch T, Twitch T_50_, and Twitch TPT produced mean CV_W_ values of 6.7%, 7.7%, 3.8%, respectively.

### 3.2 Descriptive characteristics, maximum strength, and muscle size

**FIGURE 1:**
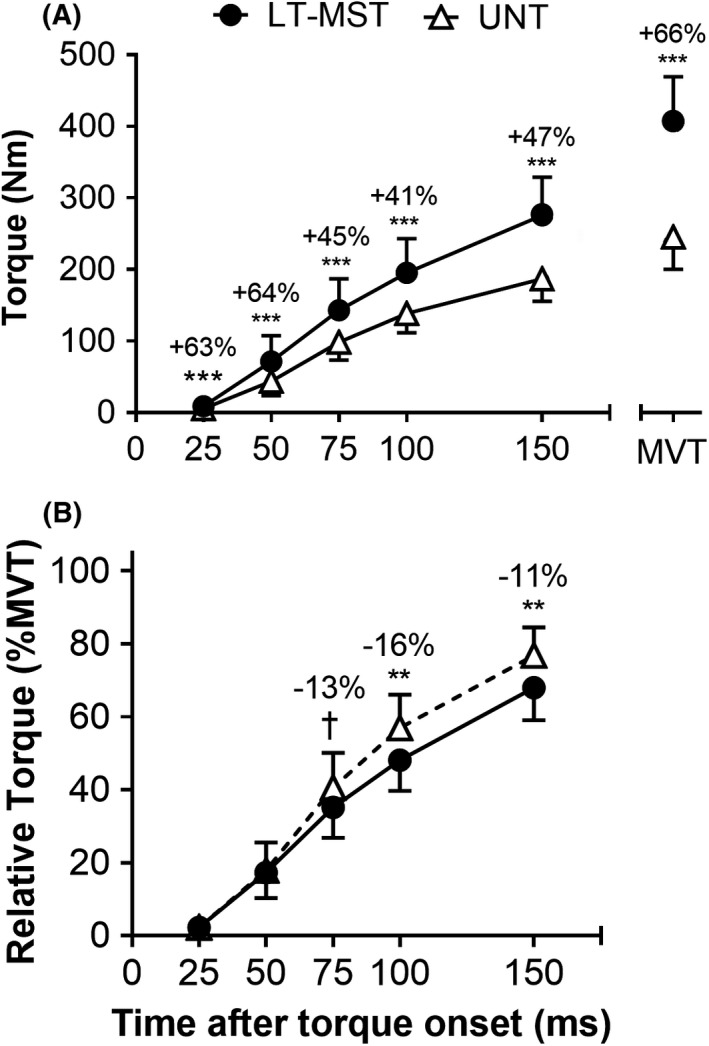
Torque in absolute (A) and relative (to maximum voluntary torque [MVT]; B) terms during voluntary explosive isometric knee extension contractions for long‐term maximum strength‐trained (LT‐MST, *n* = 14) and untrained (UNT, *n* = 49) groups. MVT recorded during maximum voluntary contractions is also shown in (A) for reference. Symbols denote differences between the two groups (independent sample t‐tests) as follows: †*p* = 0.056 (tendency), **p < 0.01; ***p < 0.001. Percentage difference between group mean values (LT‐MST relative to UNT) are shown

The LT‐MST group was younger (22 ± 2 y), taller (1.84 ± 0.06 m), and heavier (92 ± 10 kg) than the UNT group (age, 25 ± 2 y; height, 1.76 ± 0.07 m; body mass, 73 ± 9 kg; [all variables] *p *≤ 0.001). Knee extension MVT of LT‐MST (407 ± 63 Nm) was 66% greater than UNT (245 ± 45 Nm; *p* < 0.001; ES = 3.27 “Large”; Figure 1A). QACSA_MAX_ of LT‐MST (138 ± 14 cm^2^) was 54% greater than UNT (90 ± 12 cm^2^; *p* < 0.001; ES = 3.92 “Large”).

### 3.3 Absolute and relative knee extension explosive strength

**FIGURE 2:**
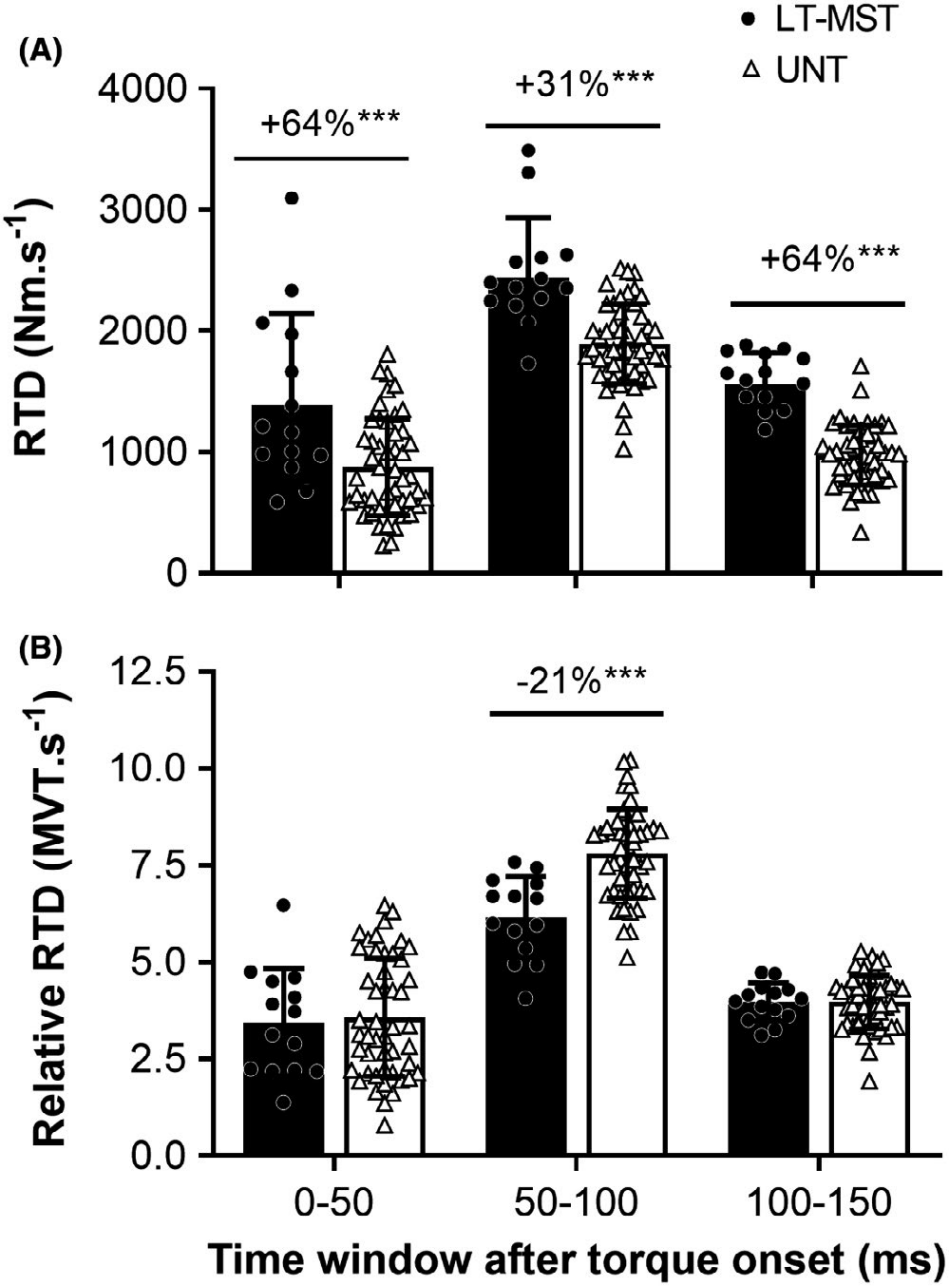
Rate of torque development (RFD) in absolute (A) and relative (to maximum voluntary torque [MVT]; B) terms for consecutive 50‐ms time windows during voluntary explosive isometric knee extension contractions for long‐term maximum strength‐trained (LT‐MST, *n* = 14) and untrained (UNT, *n* = 49) groups. Symbols denote differences between the two groups (independent sample t‐tests) as follows: ***p < 0.001. Percentage difference between group mean values (LT‐MST relative to UNT) are shown

Absolute explosive torque of LT‐MST was 41%–64% greater than UNT at 25–150 ms from torque onset ([all] *p* ≤ 0.001; 1.15 ≤ ES ≤ 2.36 [all] “Large”; Figure 1A). Relative explosive torque of the two groups was similar at 25 and 50 ms after contraction onset (0.767 ≤ *p* ≤ 0.773; ES [both] = 0.09 “Trivial”; Figure 1B), but was lower or tended to be lower for LT‐MST than UNT at 75 ms (−13%; *p* = 0.056; ES = 0.59 “Moderate”), 100 ms (−16%; *p* = 0.002; ES = 0.98 “Large”), and 150 ms (−11%; *p* = 0.001; ES = 1.11 “Large”; Figure 1B). Absolute RTD was greater for LT‐MST than UNT across all three consecutive time windows (+31–64%; [all] *p* ≤ 0.001; 1.15 ≤ ES ≤ 2.59; Figure 2A). When RTD was expressed relative to MVT, LT‐MST had lower relative RTD than UNT between 50 and 100 ms (−21%; *p* < 0.001; ES = 1.44 “Large”; Figure 2B), but similar relative RTD during the other time periods (0–50 ms, *p* = 0.774; ES = 0.09 “Trivial”; 100–150 ms, P = 1.000; ES = 0.00 “Trivial”).

### 3.4 Agonist and Antagonist EMG and neural efficacy

**FIGURE 3:**
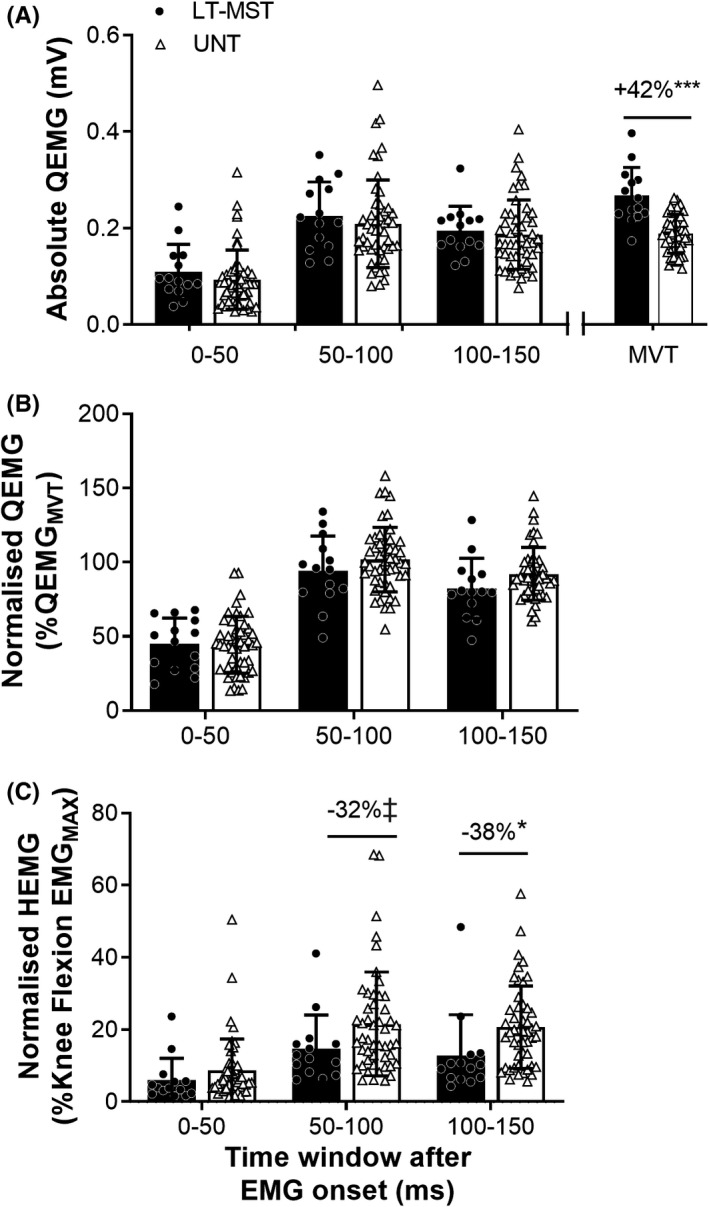
(A) Absolute agonist (Q) electromyography (EMG), (B) Normalized agonist EMG, and (C) Normalized antagonist (H) EMG during maximum voluntary torque production (MVT) and explosive contraction (0–50, 50–100, and 100–150 ms after EMG onset) of the knee extensors for long‐term maximum strength‐trained (LT‐MST, *n* = 14) and untrained (UNT, *n* = 49) groups. For absolute QEMG, root‐mean‐square amplitude was corrected for muscle‐electrode distance (ie, adiposity). Knee flexion EMG_MAX_, agonist EMG during knee flexion MVT. Symbols denote differences between the two groups (independent sample t‐tests) as follows: ‡*p* = 0.096 (tendency), *p < 0.05, ***p < 0.001. Percentage difference values denote difference in the LT‐MST group mean relative to UNT group mean

Absolute QEMG (corrected for muscle‐electrode distance) was greater during maximum voluntary torque production for LT‐MST compared to UNT (+42%; *p* < 0.001; ES = 1.75 “Large”; Figure 3A). However, absolute QEMG (corrected for muscle‐electrode distance) during explosive contractions did not differ between groups for the 0‐ to 50‐ms, 50‐ to 100‐ms, or 100‐ to 150‐ms time periods after EMG onset (0.384 ≤ *p *≤ 0.677; 0.13 ≤ ES ≤ 0.27 “Trivial” to “Small”; Figure 3A). Normalized QEMG during explosive knee extension contractions did not differ between groups across the three consecutive time periods after EMG onset (0–50 ms, *p* = 0.921, ES = 0.03 “Trivial”; 50–100 ms, *p* = 0.259, ES = 0.35 “Small”; 100–150 ms, *p* = 0.086, ES = 0.53 “Moderate”; Figure 3B). Knee extension volitional neural efficacy (ie, the voluntary T_50_/ octet T_50_ ratio) did not differ between LT‐MST (48% ± 19%) and UNT (41% ± 17%; *p* = 0.199; ES = 0.42 “Small”). Normalized HEMG during explosive knee extension contractions did not differ between groups in the early phase of explosive contraction (0–50 ms, −31%; *p* = 0.292, ES = 0.32 “Small”), but was lower or tended to be lower for the LT‐MST compared to the UNT group during the mid and later phases of explosive contraction (50–100 ms, −32%; *p* = 0.096, ES = 0.51 “Moderate”; 100‐ to 150‐ms time window (−38%; *p* = 0.026, ES = 0.69 “Moderate”; Figure 3C).

### 3.5 Intrinsic Contractile Properties

**FIGURE 4:**
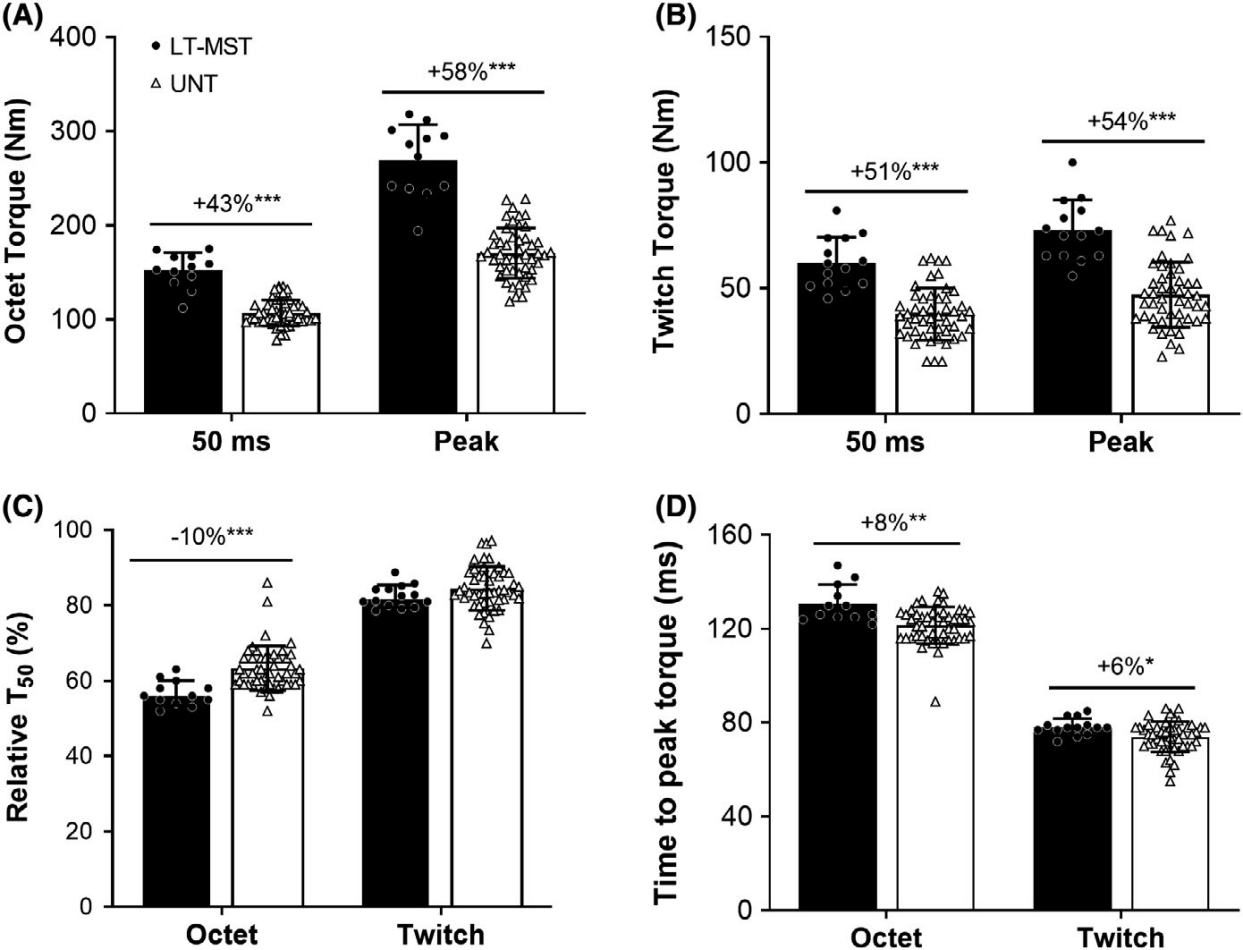
Absolute torque 50 ms after torque onset and at peak torque during evoked octet (A) and twitch (B) contractions, as well as relative torque after 50 ms (to peak torque, C) and time to peak torque (D) during these contractions for long‐term maximum strength‐trained (LT‐MST, *n* = 12) and untrained (UNT, *n* = 49) groups. Symbols denote differences between the two groups (independent sample t‐tests) as follows: *p < 0.05, **p < 0.01; ***p < 0.001. Percentage difference values denote difference in the LT‐MST group mean relative to UNT group mean

Absolute torque after 50 ms and the peak torque of the evoked octet were greater for LT‐MST than UNT (+43% and +58%; [both] *p* < 0.001; 3.07 ≤ ES ≤ 3.36 “Large”; Figure 4A). Similarly, absolute torque after 50 ms and peak torque of the evoked twitch were also greater for LT‐MST than UNT (+51% and +54%; [both] *p* < 0.001; 1.98 ≤ ES ≤ 2.01 “Large”; Figure 4B). However, Octet Relative T_50_ was lower for the LT‐MST than the UNT group (−10%; *p* < 0.001; ES = 1.14 “Large”; Figure 4C), although Twitch Relative T_50_ did not differ between LT‐MST and UNT (*p* = 0.200; ES = 0.39 “Small”; Figure 4C). Finally, both Octet and Twitch TPT were longer (ie, slower) for the LT‐MST than the UNT group (Octet TPT: +8; *p* = 0.001; ES = 1.18 “Large”; Twitch TPT: +6; *p* = 0.015; ES = 0.76 “Moderate”; Figure 4D).

## 4 DISCUSSION

The purpose of this study was to compare explosive isometric knee extension strength and key underpinning physiological factors (intrinsic contractile properties and neuromuscular activation) between cohorts with and without a history of long‐term maximum strength training (MST). The prior experience (≥3 years) of maximum strength training was confirmed by the LT‐MST group's pronounced 66% greater maximum strength. The main findings of this study were firstly that as hypothesized LT‐MST had greater absolute explosive strength throughout the rising torque‐time curve compared to their untrained counterparts (+41–64%) and this appeared to be primarily due to larger muscle size (QACSA_MAX_ +54%) given the equivalent neuromuscular activation observed between groups during explosive contractions (measured with surface EMG and neural efficacy). Secondly, in partial agreement with our hypothesis the LT‐MST group had lower relative RTD during the mid/fastest rising phase of contraction (RTD 50–100 ms) compared to the UNT cohort that led to lower relative explosive strength from 100 ms onward (ie, T_100_ −16%, T_150_ −11%). Thirdly, as hypothesized we also found LT‐MST had slower evoked contractile properties, specifically a longer time to peak twitch and octet torque and a lower Octet Relative T_50_, compared to the untrained cohort. Overall, according to these findings LT‐MST individuals have a substantially enhanced absolute explosive strength, but lower relative ability to express the available torque generating capacity during the mid and late phases of explosive contraction compared to that of untrained individuals likely due to the slower contractile properties of LT‐MST individuals.

Consistent with our hypothesis, LT‐MST individuals had greater absolute voluntary explosive strength than untrained controls throughout the first 150 ms of contraction (41%–64%). Previous studies have reported equivalent^21^ and greater^22^ absolute explosive strength of LT‐MST groups compared to controls, although both of these studies had more modest differences in maximum strength (35% and 23%, respectively) that might indicate less well‐trained groups than we have studied. While agonist neuromuscular activation has been found to be a major determinant of explosive torque production particularly during the early phase of contraction,^3^, ^33^, ^34^ in the current experiment, there were no between group differences in agonist activation. In contrast, evoked explosive torque during twitch and octet contractions was markedly higher in LT‐MST than UNT (Twitch T_50_ +51%; Octet T_50_ +43%). The evoked octet in particular has been found to drive the muscle at its maximum possible rate of force development,^29^, ^30^ and thus, the current study shows a substantially greater intrinsic contractile capacity for explosive strength of LT‐MST individuals. Both the greater contractile and voluntary explosive strength of the LT‐MST group are likely due to their larger muscle size (ACSA_MAX_ +54%) as this is a known important predictor of voluntary (T_100_ and T_150_) and evoked (T_50_) explosive strength.^17^

The findings of the present investigation suggest that multiple years of maximum strength training increases absolute explosive strength across the entire rising torque‐time curve, an observation in contrast to several short‐term (3–12 weeks) longitudinal maximum strength training interventions where increases in absolute explosive strength did not occur,^15^ only increased in the late phase of contraction (ie, 150 ms after contraction onset^12^, ^13^), or even decreased in the early phase of contraction (ie, 50 ms) with no change thereafter.^14^ The opposing absolute explosive strength results of the current investigation compared to previous short‐term longitudinal maximum strength training studies are likely explained by the modest hypertrophy in those studies compared to the large difference within the current investigation (+54%). In addition, similar to the current investigation the short‐term longitudinal studies of maximum strength training have also found no changes in explosive phase neuromuscular activation^12^, ^13^, ^14^ and a slowing of the contractile properties^12^ that might negate any benefit of modest hypertrophy on absolute explosive strength. Antagonist (hamstrings) EMG was lower for LT‐MST compared to UNT in the late phase of contraction (100–150 ms). This observation of reduced co‐activation particularly in the late phase of explosive contraction is in accordance with our previous finding of lower co‐activation of LT‐MST compared to UNT individuals during sustained contractions.^35^

Explosive torque (normalized to MVT) was lower for LT‐MST than UNT from 100 ms onward (−11% to −16%), indicating a lower relative ability to utilize the available torque (defined by MVT) explosively. This finding of lower relative explosive torque of LT‐MST was consistent with our hypothesis and our previous short‐term longitudinal maximum strength training studies.^12^, ^13^, ^14^ This discrepancy in relative explosive torque in the later phase of contraction arose due to the lower relative RTD of LT‐MST from 50 to 100 ms, with equivalent relative RTD during other periods. LT‐MST also had lower evoked relative explosive torque (relative octet T_50_ −10%) and a longer/slower time course of evoked contractile properties (ie, time to peak torque: octet +8%; and twitch +6%) than UNT participants, both measures that are independent of torque amplitude. These findings are complimentary as a longer time to peak tension would be expected to also involve production of lower relative torque at a fixed time point during the rising/explosive phase of contraction (eg, relative octet T_50_), and therefore, the concomitant nature of these findings adds veracity to the observation of slower intrinsic contractile properties of the muscle after LT‐MST. Furthermore, these between‐group differences are consistent with previously observed reductions in evoked relative explosive torque (relative octet T_50_ −8%) and longer/slower time to peak torque (octet +7%; and twitch +5%) after short‐term longitudinal maximum strength training.^12^ Moreover, the intrinsic contractile capacity for explosive torque, assessed by octet T_50_/octet RTD_0‐50_, has been found to be the primary determinant of voluntary RTD 50–100 ms, when both variables are expressed in either absolute or relative terms.^3^ Therefore, the lower evoked relative explosive torque (ie, relative octet T_50_ −10%) of LT‐MST observed in the current experiment likely explains their lower relative voluntary RTD from 50 to 100 ms and consequently their lower voluntary explosive torque from 50 ms onward.

Overall, based on the short‐term studies and the current experiment, it may take many months of maximum strength training to significantly enhance explosive strength via the progressive accumulation of muscle growth. Therefore, some element of explosive strength training may be needed to produce short‐/medium‐term increases in explosive strength by improving explosive neuromuscular activation and thus the ability to express the available torque generating capacity explosively (ie, enhanced relative explosive strength) leading to higher absolute explosive strength. However, given sufficient maximum strength training duration the enhanced muscle size appears to improve the contractile capacity for explosive force production such that, even with no improvement in explosive neuromuscular activation, voluntary explosive strength is enhanced.

The slower intrinsic contractile properties of the LT‐MST group relative to the untrained cohort were likely due to decreased myosin heavy‐chain type IIX expression following prolonged maximum strength training.^36^, ^37^, ^38^ Consistent with this idea individuals who have completed long‐term maximum strength training have previously been documented to possess lower myosin heavy‐chain type IIX expression than those who are untrained.^39^ Indeed, when comparing the contractile properties of isolated rodent hindlimb muscles with high (extensor digitorum longus) and low (soleus) myosin heavy‐chain type IIX expression,^40^ those muscles with higher myosin heavy‐chain type IIX expression display faster times to peak twitch force.^41^ To the best of our knowledge, the current study is the first to document slower intrinsic contractile properties, evidenced by a longer/slower timecourse and lower relative evoked explosive torque, of a cohort with multiple years of maximum strength training experience compared to an untrained population.

The present study provides some novel insight into how long‐term maximum strength training likely influences explosive strength and the underpinning contractile and neuromuscular activation characteristics, given the lack of equivalent longitudinal or cross‐sectional studies in the literature. However, the limitations of the present study must still be considered. The cross‐sectional design of the study inherently provides weaker evidence than a longitudinal study design and means that the adaptations due to training cannot be entirely separated from the innate characteristics of the groups. Longer duration longitudinal RT studies (eg, 12–18 months), ideally with repeated measurements throughout the intervention period, are needed to better understand the long‐term adaptations to RT. Within the current study, more detailed measurements of evoked contractile properties and motor unit behavior (ie, via high density EMG) would also have furthered our understanding of how maximum strength training influences explosive strength. Additionally, while participants within the LT‐MST group were recruited based on having a history of ≥3 years of resistance training focused on developing maximal strength, they did not complete a uniform maximum strength training program (ie, standardized exercises, repetitions, sets, and load) and this may have introduced some variability within this group. The smaller sample size of the LT‐MST group due to this being a more select group was also a limitation of the study. A larger LT‐MST group sample may have clarified some of the borderline results (ie, statistical tendencies) we observed. With respect to the evoked contractions, it is not possible to verify that all the muscle fibers in every participant were activated by electrical stimulation, although the carefully calibrated supramaximal stimulation of the femoral nerve would be expected to deliver evoked action potentials to all alpha motoneuron axons and thus all knee extensor muscle fibers.

In conclusion, individuals who have completed multiple years of maximum strength training possessed not only greater maximum but also absolute explosive torque production compared to that of an untrained population. The greater absolute explosive strength across the rising torque‐time curve of the LT‐MST group, relative to untrained, was attributable to greater evoked explosive torque, in turn due to larger muscle size, rather than any differences in agonist neuromuscular activation during explosive contractions. Interestingly, LT‐MST had lower relative (to MVT) explosive torque production and thus were less able to utilize the available torque generating capacity within the first 150 ms of contraction. This deficit in relative explosive strength appeared to be underpinned by lower evoked relative explosive torque and slower contractile properties quadriceps (measured during evoked twitch and octet contractions), likely as a result of reduced expression of type IIX fibers.

## 5 PERSPECTIVE

Some short‐term studies have indicated a high level of specificity in the adaptations to resistance training. However, the effects of several years of training for maximum strength on explosive strength and associated intrinsic contractile properties remain to be elucidated and may inform the specificity/commonality of different types of strength following resistance training. The enhanced explosive strength after long‐term training for maximum strength in the current study reinforces the training commonality, rather than the specificity, of these types of strength. However, the findings also support our previous evidence that resistance training slows the intrinsic contractile properties and appears to lower the ability to express the available torque capacity explosively (ie, relative explosive strength). Thus, despite the short‐term evidence for highly training specific strength changes, this study provides greater clarity that given sufficient time for morphological adaptations (ie, marked changes in muscle size) there is considerable commonality between maximum and explosive strength adaptations that may inform resistance training prescription.

## 6 GRANTS

Part of this study was supported by a grant (reference 20194) awarded to Professor Folland from the Arthritis Research UK Centre for Sport, Exercise, and Osteoarthritis.

## CONFLICT OF INTEREST

The authors declare that there is no conflict of interest, that no companies or manufacturers will benefit from the results of the study, and that the results of the study are presented clearly, honestly, and without fabrication, falsification, or inappropriate data manipulation.

## Data Availability

The data that support the findings of this study are available from the corresponding author upon reasonable request.
